# Sequenced genomes and chromosome mapping illuminate key aspects of satellite DNA biology in *Drosophila gouveai* and *D. borborema* (*buzzatii* cluster, *repleta* group)

**DOI:** 10.1590/1678-4685-GMB-2025-0133

**Published:** 2025-12-08

**Authors:** Anna C. Rossi, Ana M. Laborne, Dora Y. Barrios-Leal, Maura H. Manfrin, Gustavo C.S. Kuhn

**Affiliations:** 1 Universidade Federal de Minas Gerais, Instituto de Ciências Biológicas, Departamento de Genética, Ecologia e Evolução, Belo Horizonte, MG, Brazil.; 2 Universidade de São Paulo, Faculdade de Medicina de Ribeirão Preto, Departamento de Genética, São Paulo, SP, Brazil.; 3 Universidade de São Paulo, Faculdade de Filosofia, Ciências e Letras de Ribeirão Preto, Departamento de Biologia, São Paulo, SP, Brazil.

**Keywords:** Satellite DNA, centromeres, heterochromatin, Drosophila

## Abstract

Satellite DNAs are abundant components of the genomes of many eukaryotic species. They are composed of long and rather homogeneous arrays of tandem repeats that are typically located at the heterochromatin. They may contribute to the structural organization and regulatory dynamics of the genome. However, they evolve rapidly between species and changes in their sequences and abundance may contribute to the process of speciation. Here we used Illumina genomic sequencing raw data and the TAREAN bioinformatic tool to identify and characterize the most abundant satDNAs present in two sister species from the *buzzatii* cluster (*repleta* group): *D. gouveai* and *D. borborema*. We found five satDNAs, two are reported in these species for the first time (CDSTR138 and CDSTR230), two have already been reported (pBuM and DBC-150) and one is described here for the first time (CDSTR8). These satDNAs differ in both quantitative and qualitative terms between the two species. Most notoriously, the pBuM satDNA was found in *D. gouveai,* but it is virtually absent in *D. borborema*, despite their relatively recent divergence (<0.5My). We mapped these satDNAs to the chromosomes and found that most of them are located near or at the centromeres, with overlapping distribution in several locations.

Repetitive DNAs make up the major proportion of the genome of many eukaryotic species (Biscotti et al., 2015). Satellite DNAs (satDNAs) are among the most abundant components of this fraction, yet they remain one of the least understood. SatDNAs are included in the highly repetitive fraction of the genome (>1,000 copies) and are made of hundreds to thousands of tandem repeats forming large arrays typically located in the heterochromatic parts of the chromosomes (Charlesworth et al., 1994). In many eukaryotic species, satDNAs are often seen as the main components of the heterochromatin. Consequently, variations in the amount of heterochromatin between chromosomes and between species are correlated with changes in the abundance of satDNAs (Kuhn et al., 2009). Although satDNA repeats do not encode proteins, they may contribute to the structural organization and regulatory dynamics of the genome. For instance, in many eukaryotes, satDNAs are major components of centromeric regions (Henikoff et al., 2001), thereby participating in the critical role of kinetochore assembly and proper chromosome segregation during cell divisions. On the regulatory side, increasing evidence shows the involvement of satDNAs in modulating gene expression and the global heterochromatin structure (Feliciello et al., 2021; Sneideman and Meller 2021). Nevertheless, they exhibit rapid evolutionary dynamics and for this reason they often vary significantly even among closely related species. SatDNA divergence between populations has been regarded as a component of the speciation process (Ferree and Barbash, 2009). Given the fast evolutionary dynamics of satDNAs, studies leading to their characterization need to be conducted on a species-by-species basis. 

Since the discovery of satDNAs in the early 1960`s, *Drosophila* species have served as important models for understanding various aspects of satDNA biology. However, most studies are concentrated in the *Sophophora* subgenus, even though more than 80% of all *Drosophila* species belong to the *Drosophila* subgenus (O’Grady and DeSalle 2018). In particular, very little is known about satDNAs in the New World *repleta* group (*Drosophila* subgenus) that includes more than 100 described species (Oliveira et al., 2012). 

Within the *repleta* group, significant efforts have been particularly made towards the study of satDNAs from a monophyletic group of species called *buzzatii* cluster, which comprises seven species: *D. buzzatii*, *D. koepferae*, *D. antonietae*, *D. serido*, *D. seriema*, *D. gouveai* and *D. borborema*. Except for *D. buzzatii* that became subcosmopolitan, all other species are endemic to South America (Manfrin and Sene, 2006). Divergence times between species range from less than 0.5My to 4My (Franco and Manfrin, 2012; Oliveira et al., 2012; Moreyra et al., 2023), what makes this group particularly amenable to study satDNA evolution at recent time scales. While the availability of sequenced genomes of *D. buzzatii*, *D. seriema*, *D. serido* and *D. antonietae* has provided several new insights on the satDNA collection of these species, very little is known about the satDNAs from *D. gouveai* and *D. borborema*, which together with *D. serido* and *D. seriema* belong to a phyletic lineage within the cluster known as “*Drosophila serido* haplogroup” (Franco and Manfrin 2012) (Figure S1). Previous studies in *D. gouveai* and *D. borborema* relied on the identification of satDNAs by genomic DNA digestions and subsequent mapping in mitotic chromosomes. The basic karyotype of species from the *buzzatii* cluster consists of one pair of acrocentric sex chromosomes (XX or XY), four pairs of acrocentric autosomes of similar size and one pair of microchromosomes. Heterochromatin is concentrated in the centromeric region of the four acrocentric autosomes and occupies almost the entire length of the microchromosome and the Y chromosome. The acrocentric X chromosome contains pericentromeric heterochromatin that extends up to half the size of its long arm (Baimai et al., 1983; Kuhn et al., 2007). In *D. gouveai*, the pBuM satDNA was detected in the (peri) centromeric regions of the X chromosome, in two acrocentic pairs (out of four) and in the microchromosome pair (Kuhn *et al.,* 2008; 2009). The DBC-150 satDNA was found restricted to the microchromosomes (Kuhn *et al.,* 2007). Surprisingly, while DBC-150 was also exclusively detected in the microchromosomes of *D. borborema* (Kuhn *et al.,* 2007), pBuM was not detected in any other chromosome, revealing drastic changes in pBuM copy number between the two species (Kuhn *et al.,* 2008). 

In the present work, we aimed to revisit the satDNA content of *D. gouveai* and *D. borborema* by using Illumina paired-end sequencing raw data and the TAREAN bioinformatics tool to identify and characterize the most abundant satDNAs in these species and to determine their chromosome location by *in situ* hybridization on mitotic chromosomes. 

For *D. gouveai*, we used sequencing reads from a population of Altinópolis (strain H6; São Paulo state Brazil), while for *D. borborema*, we used sequencing reads from two populations collected in the state of Bahia (Brazil): Morro do Chapéu e Milagres, hereafter referred to as *D. borborema A* and *B*, respectively (see Franco and Manfrin 2012 for precise geographic location). The sequencing data for *D. gouveai* and *D. borborema B* was generated with Illumina HiSeq 2500 by the group of one of the co-authors of this work (M.H. Manfrin), while the sequencing data from *D. borborema A* is publicly available in GenBank (SRX18184288) and was also generated with Illumina HiSeq 2500 (Moreyra et al., 2023). 

All *in silico* analyses were performed on the Galaxy Platform (Afgan et al., 2018) using the RepeatExplorer2 pipeline (Novák et al., 2020), which includes an integrated version of the TAREAN tool for satellite DNA identification based on cluster graph topology. Read quality filtering, preprocessing and clustering parameters followed Laborne et al. (2024). Putative satDNAs with more than 1,000 copies in at least one genome were selected for further analysis (Table 1), in accordance with classical satDNAs definitions (Tautz, 1993; Charlesworth et al., 1994). Consensus sequences were queried against NCBI, Repbase, and FlyBase databases using BLASTn to assess similarity with known repetitive elements. 

**Table 1 -:** General characteristics of putative satellite DNAs identified by TAREAN in *D. gouveai* and *D. borborema*.

Genome	Repeat Explorer Cluster	Satellite DNAs	Estimated Copy Number	Genomic DNA Proportion (%)	Satellite Probability (%)	Consensus Length (bp)
*D. gouveai*	1	CDSTR230	4,461	0.578	0.992	230
	5	IGS-109TRª	9,837	0.469	9.55e-01	86
	6	CDSTR138	6,221	0.468	0.981	136
	8	CDSTR8	99,450	0.442	0.933	24
	9	pBuM-2	1,995	0.412	0.992	370
	30	DBC-150	2,537	0.216	0.933	149
	100	CDSTR198ᵇ	603	0.063	0.0661	188
*D. borborema A*	1	CDSTR8	403,200	1.792	0.979	16
	20	CDSTR230	1,910	0.262	0.994	245
	32	CDSTR138	2,348	0.177	0.994	138
	40	DBC-150	2,089	0.133	0.985	112
	17	CDSTR198ᵇ	2,337	0.269	0.815	208
*D. borborema B*	2	CDSTR8	146,250	0.650	0.985	32
	7	CDSTR230	2,571	0.350	0.986	245
	24	IGS-109TRª	4,395	0.209	0.853	86
	25	CDSTR138	2,739	0.200	0.986	138
	30	DBC-150	3,214	0.180	0.994	112
	38	CDSTR198ᵇ	1,820	0.110	0.941	178

ª = rDNA (see Laborne et al., 2024 for details).

ᵇ = minisatellite (see Kuhn et al., 2021 and Laborne et al., 2024 for details).

Mitotic chromosomes were obtained from neuroblasts of third instar larvae of *D. gouveai* (strain H6), following Baimai (1977) and Ashburner (1989), with modifications as described in Laborne et al. (2024). Probes were labeled with digoxigenin-11-dUTP or biotin-11-dUTP via nick translation (Roche DIG- and Biotin-nick translation mix) except for CDSTR8, which was manufactured as a 5’-biotin-labeled probe (Síntese Biotecnologia LTDA) containing five tandemly repeated monomers of 8 bp each, reaching 40 bp in total. Hybridizations were performed following Dias et al. (2014), with minor modifications, including a 1 min 15 s treatment with 0.07 M NaOH. Chromosomes were counterstained with DAPI in Antifade (SlowFade, Invitrogen) and visualized using a Zeiss Axio Imager A2 epifluorescence microscope equipped with an AxiocamMRm camera. Images were captured with Axiovision and edited in Adobe Photoshop^®^. 

After manual inspection of all putative satDNAs revealed by TAREAN, we found that *D. gouveai* and *D. borborema* share four satDNAs, DBC-150, CDSTR138, CDSTR230 and CDSTR8, while the fifth satDNA, pBuM, was found only in *D. gouveai* (Table 1).

The DBC-150 is a previously described satDNA in species from the *buzzatii* cluster with monomers falling in three main size variants, 46 bp, 112 bp and 150 bp long (Kuhn et al., 2007; 2009). This satDNA has been previously identified in *D. gouveai* and *D. borborema* based on genomic DNA digestions with restriction enzymes or PCR amplification (Kuhn *et al.,* 2007). Here, we found monomers of 112 bp and 150 bp in both species, but TAREAN returned a consensus sequence of 112 bp for both populations of *D. borborema*, and a consensus sequence of 150 bp for *D. gouveai*. 

The CDSTR138 is a satDNA with monomers around 138 bp long. This satDNA has been previously identified in other species from the *buzzatii* cluster and it is here reported in *D. gouveai* and *D. borborema* for the first time. TAREAN returned a CDSTR138 consensus sequence of 136 bp for *D. gouveai* and 138 bp for both populations of *D. borborema*.

The CDSTR230 has been recently described in *D. serido* and *D. antonietae* (Laborne et al., 2024) and presents monomers from 197 to 230 bp long. For *D. gouveai*, TAREAN returned a consensus sequence of 230 bp, while for both populations of *D. borborema*, the consensus sequence was 245 bp.

The CDSTR8 has not been previously detected in any *Drosophila* species and is described here for the first time. For *D. gouveai*, TAREAN returned a consensus sequence of 24 bp, while for *D. borborema,* the consensus sequence was 16 bp for *A* and 32 bp for *B*. However, a more detailed sequence analysis revealed that this satDNA is actually made of 8 bp basic repeats, organized in both monomeric form (i.e. 8 bp monomers) or as high-order repeats (up to five 5 monomers). The size of this satDNA is unusual compared to other known satDNAs described in species from the *repleta* group, which usually falls in the range of 100 to 400 bp (Kuhn et al., 2021).

The pBuM satDNA has been previously identified in several species from the *buzzatii* cluster and has two major variants: pBuM-1 with monomers 190 bp long and pBuM-2 with monomers 370 bp long. In *D. gouveai*, TAREAN returned only pBuM-2 repeats, confirming that this variant is the most predominant in this species (Kuhn and Sene, 2005; Kuhn *et al.,* 2008). For *D. borborema*, we did not detect pBuM in any repetitive cluster returned by TAREAN, confirming previous studies that suggested that this satDNA is virtually absent in this species (Kuhn and Sene, 2005; Kuhn *et al.,* 2008).

All satDNA consensus sequences returned by TAREAN are shown in Data S1, and the nucleotide alignments between the consensus sequences of *D. gouveai* and *D. borborema* are shown in Figure S2. Besides the five satDNAs reported above, it is worth mentioning that two other tandemly repeated elements, CDSTR198 and IGS-109TR, have also initially passed our criteria for putative satDNAs. However, previous studies revealed that CDSTR198 is a minisatellite DNA, organized as small arrays spread in the euchromatin (Lima et al., 2017; Laborne et al., 2024), while IGS-109TR is an intergenic spacer of ribosomal genes (Laborne *et al.,* 2024). For these reasons, these repetitive elements were not included in further analysis. 

Taking all satDNAs together, the estimated contribution of satDNAs for total genomic DNA is approximately 2% for *D. gouveai* and 2 to 3% for *D. borborema*, depending on the population (Figure 1). These values can be considered low for *Drosophila* but are similar to the ones obtained for other species from the *buzzatii* cluster or *repleta* group (<5 %) (Kuhn et al., 2021). It remains to be investigated whether such low satDNA content is a consequence of selective constraints or stochastic events. Between the two species, there are some satDNA quantitative differences. For example, CDSTR230 is the most abundant satDNA in *D. gouveai*, while CDSTR8 is the most abundant satDNA in *D. borborema*; pBuM was only found in *D. gouveai*. Interestingly, CDSTR8 revealed a difference between the strains from the two populations of *D. borborema*, 1.8% for *A* and 0.7% for *B*. This result contrasts to the low levels of genetic differentiation among *D. borborema* populations revealed by mtDNA (Franco and Manfrin, 2012). Since our sample was small (one sequenced genome from each population), it remains to be investigated if this difference represents an intra or inter-population variation. 

**Figure 1 -:**
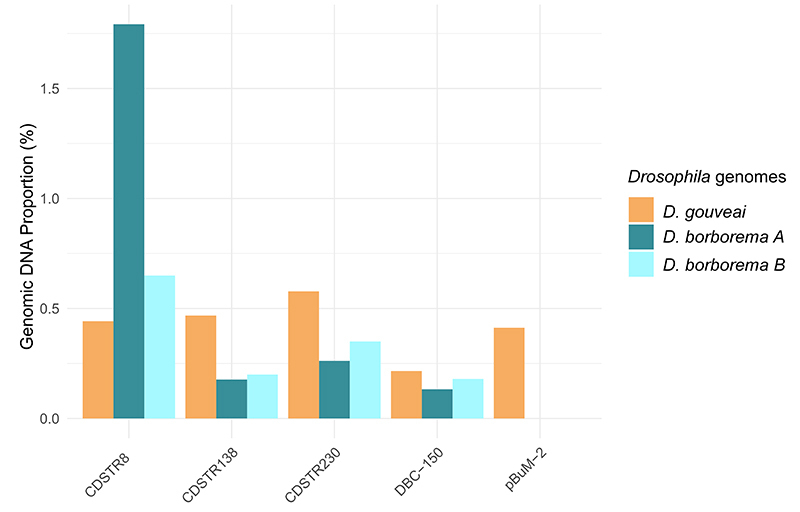
Genome proportion of satellite DNAs identified *D. gouveai*, *D. borborema A* and *D. borborema B.*

FISH on the mitotic chromosomes of *D. gouveai* revealed that all satDNAs localize in the heterochromatic regions of the chromosomes. The novel satDNA CDSTR8 localized in the centromeric region of the four pairs of acrocentric chromosomes and in the centromeric region of the X chromosome (Figure 2a). The CDSTR230 satDNA localized in the microchromosomes, in the β-heterochromatin region (transition between α and α-heterochromatin) of the X chromosome, and in two separate loci on the long arm of the Y chromosome (Figure 2a). In *D. serido* and *D. antonietae*, this satDNA was found restricted to the Y chromosome (Laborne et al., 2024). Considering the phylogeny of these species (Figure S1), this result suggests a dispersal of CDSTR230 from the Y chromosome to other chromosomes in *D. gouveai*.

**Figure 2 -:**
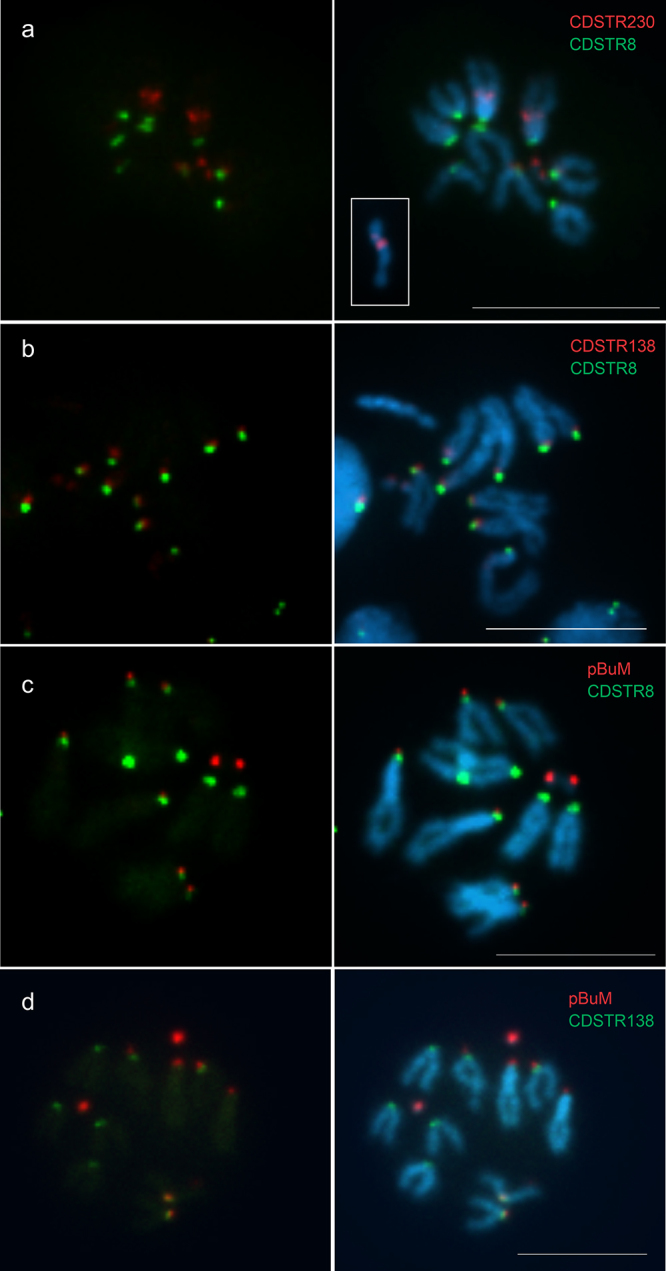
Fluorescent *in situ* hybridization in *D. gouveai* mitotic chromosomes. Probes are indicated in red or green. Scale bars represent 5µM.

The CDSTR138 satDNA localized in the centromeric region of the four pairs of acrocentric chromosomes in *D. gouveai* (Figure 2b). In *D. seriema*, *D. serido* and *D. antonietae*, it has also recently been discovered that this satDNA is most likely the main centromeric component of these four pairs of acrocentric chromosomes (Laborne et al., 2024). 

Since our results indicated possible co-localization of pBuM, CDSTR8 and CDSTR138 in the centromeric regions of the four acrocentric chromosomes, we performed double-FISH experiments with probes for pBuM/CDSTR8 (Figure 2c) and pBuM/CDSTR138 (Figure 2d). The pBuM satDNA seemed to occupy a more distal position compared to CDSTR8 and CDSTR138 satDNAs (Figures 2c and 2d). Finally, a double-FISH with probes for CDSTR8 and CDSTR138 revealed that both satDNAs colocalize in the centromeric regions of the four acrocentric pairs, but CDSTR8 seemed to extend to more distal locations in the two acrocentric pairs that lack the pBuM satDNA (Figure 2b). All the results obtained here with FISH using satDNA probes in *D. gouveai* are summarized in Figure 3.

**Figure 3 -:**
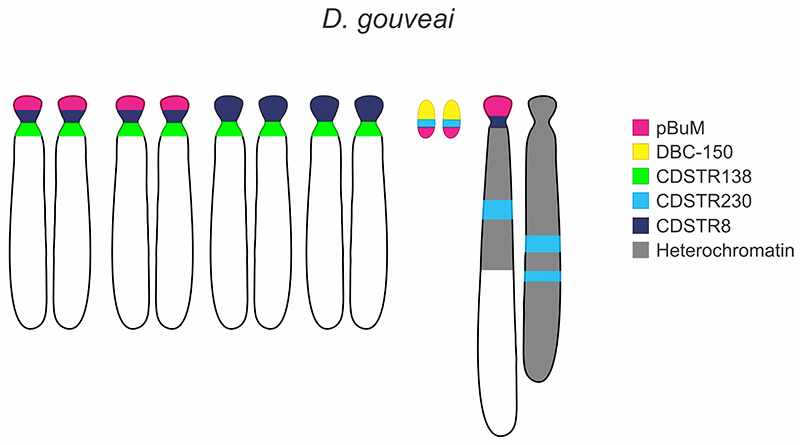
Idiogramatic representation of the metaphase karyotypes of *D. gouvea*i with the localization of the five satDNAs identified in the present and previous works.

Among the species of the *buzzatii* cluster, *D. borborema* is the one for which our knowledge about satDNA content and chromosome distribution remains the most limited. This is because the only previously known satDNA in this species was DBC-150, which was located exclusively in the microchromosome pair (Kuhn et al., 2007). Unfortunately, *D. borborema* is very difficult to keep under laboratory conditions, so that we had no strains available for chromosome preparations. However, given the close phylogenetic relationship between *D. borborema* and *D. gouveai* (Figure S1), and the satDNA data presented here and previous works, we predict that the centromeres of *D. borborema* are probably composed by CDSTR8 and/or CDSTR138. 

In conclusion, the use of sequenced genomes of *D. gouveai* and *D. borborema* contributed significantly to our understanding of the satDNA content of the two species, revealing the presence of two previously not detected satDNAs, CDSTR138 and CDSTR230, and a novel one, named CDSTR8, which also showed evidence of high-order organization. The localization of these satDNAs, together with the ones where chromosome location has been previously verified (pBuM and DBC-150), provided us with a much better picture of the relationships between satDNAs and heterochromatin, and between satDNAs and centromeres. In particular, we found that the centromeres of the four acrocentric chromosomes are potentially made by more than one satDNA. Future studies need to address the question whether the centromeric protein Cid (*Drosophila* CenH3), which is a marker for centromere identification (Henikoff et al., 2001), binds to one or more satDNAs in the centromeric region. Altogether, the data presented here fill important gaps that help elucidate key aspects of satDNA biology in the two species and in the *buzzatii* cluster in general. 

## Supplementary material

The following online material is available for this article:

Data S1 - Consensus sequences of the satDNAs present in *D. gouveai* and *D. borborema*.

Figure S1 - Phylogenetic relationships among species of the *Drosophila buzzatii* cluster (adapted from Franco and Manfrin 2012).

Figure S2 - Nucleotide alignments with satDNA consensus sequences from *D. gouveai* and *D. borborema*.

## Data Availability

 The sequencing data of *Drosophila borborema* A are publicly available in GenBank under the accession number SRX18184288. Additional data that support the findings of this study will be provided upon request.
