# Supplementary material for: Sequenced genomes and chromosome mapping illuminate key aspects of satellite DNA biology in Drosophila gouveai and D. borborema (buzzatii cluster, repleta group)
Source: Genet Mol Biol. 2025 Dec 8;48(4):e20250133. doi: 10.1590/1678-4685-GMB-2025-0133 (PMC12697916; doi:10.1590/1678-4685-GMB-2025-0133)
Supplement: Data S1 - [file 1415-4757-GMB-48-04-e20250133-s1.pdf]

Genetics and Molecular Biology
https://doi.org/10.1590/1678-4685-GMB-2025-0133

Supplementary Material to "Sequenced genomes and chromosome mapping illuminate key aspects of satellite DNA biology in *Drosophila gouveai* and *D. borborema* (*buzzatii* cluster, *repleta* group)"

```
>CDSTR8_Dgouveai

CATCGTTACGTCAATACATCAATA

>CDSTR8_Dborborema

CATCGATACATCAATA

>CDSTR230_Dgouveai

ACTGAATGTGTGTGCAGAAATCAGTGTTGTCTTGTTTTAAACAGTGTCATCTAATTTATATTCAGTAATTGACTTAATATATCACATCTTTTACCTGTTTG
CTTCTAGTAACGTTCCGCCTACTCTTATTTATATAAGCTTCTGGCAACTATTCTTTTATATTATTTATTTATGTAATTAAAGCAGGGTTGTTTTCACTTGT
CTTAATTTTTGCTTTTCCACTACTTTTT

>CDSTR230_Dborborema

ACTGAATGTGTGTACACAAATCAGGGTTGTCTTGTTTTATATTCATTTTTCTAAACAGTGTTATCTAATTTATATTCAGTAATTGACTTAATATATCACAT
CTTTTACCTGTTTGCTTCCAGCAACTTTCCGCCTACTCTTATTTATATAAGATTCCGGCAACTATTCTTTTATATTATTTATTTATGTAATTAAAGCAGGG
TTGTTTTCACTTGTCTTAATTTTTGCTTTTCCACTACTTTTTT

>CDSTR138_Dgouveai

CAACTGTTGATTTTTGTATATGGAATACGAATAAAATCAAATTTATAGTGTAGAGCAGTATATATGCTCCGATTTACTATGAACATAATCTTAGTTTGATT
TTTATCATGTATAGTGAGTATAGGCATACCTGGGC

>CDSTR138_Dborborema

CAACTGTTGATTTTCGTATATAGAATACGAACAAAATCAAATATATAGTGTAGACGAGTATATTTGCGCCGATTTACCATGAACATAACACTAGTTTGATT
TTGTATTCATTTATAGTGAGTATAGGCATACCTGGGC

>pBuM_Dgouveai

TACTGTCGCAAAAACCGGCCTGTGCGAAAAAAATTGAATATTTTCGAGGTTTAACAAGCTATAACCGGAGTATTTTTCATTCGACTTCTTTATGGCATACC
ATTTCAGAAGCGTCCTTTATCGGAGAACTCAGATATATCTAAGGATCTGGCATGGTCTAAGAACTTTCCGAAATATTCACATAATTCCATAAAAATATGAC
CAATTGAACTTTAAATGGCTCTAGCTAGGTTATCCTTTGTCCGATTTTCAGCTCAACTATACTTTTTGAATCCACATAGACAGTTCTTTTGATATAAACAC
AAACATTTTTTTCGAAAAAATTTTCATTTTTGCCATTTTTACCATCTTAGGTAACGTAAACCCTCTT

>DBC-150_Dgouveai

GCGGTCCGGGTACTGCCCAGATCCAACACCCGCGTATAACGTTCGGCGTAGAATTTTGGGTGTCACCGCGTATTACCGCTGGGCCGGGGTATTGGTCAAGC
CCCAGTCACATATGTATGTAGCAGGAGGCGGCCGGGTAATGCGAAGTC

>DBC-150_Dborborema

GCGGTACGGATATATACCGGATACTGTCCACGTATTTCCGTTGGGCCGGGTTATAGGTCAAGCCCCAGTCACATATGTATGTAGCAGGAGGCGGCCGGGTA
TTGCCGGAATC
```

Data S1 - Consensus sequences of the satDNAs present in *D. gouveai* and *D. borborema*.
