# Supplementary material for: Sequenced genomes and chromosome mapping illuminate key aspects of satellite DNA biology in Drosophila gouveai and D. borborema (buzzatii cluster, repleta group)
Source: Genet Mol Biol. 2025 Dec 8;48(4):e20250133. doi: 10.1590/1678-4685-GMB-2025-0133 (PMC12697916; doi:10.1590/1678-4685-GMB-2025-0133)
Supplement: Figure S2 - [file 1415-4757-GMB-48-04-e20250133-s3.pdf]

Genetics and Molecular Biology
https://doi.org/10.1590/1678-4685-GMB-2025-0133

# Supplementary Material to "Sequenced genomes and chromosome mapping illuminate key aspects of satellite DNA biology in *Drosophila gouveai* and *D. borborema* (*buzzatii* cluster, *repleta* group)"

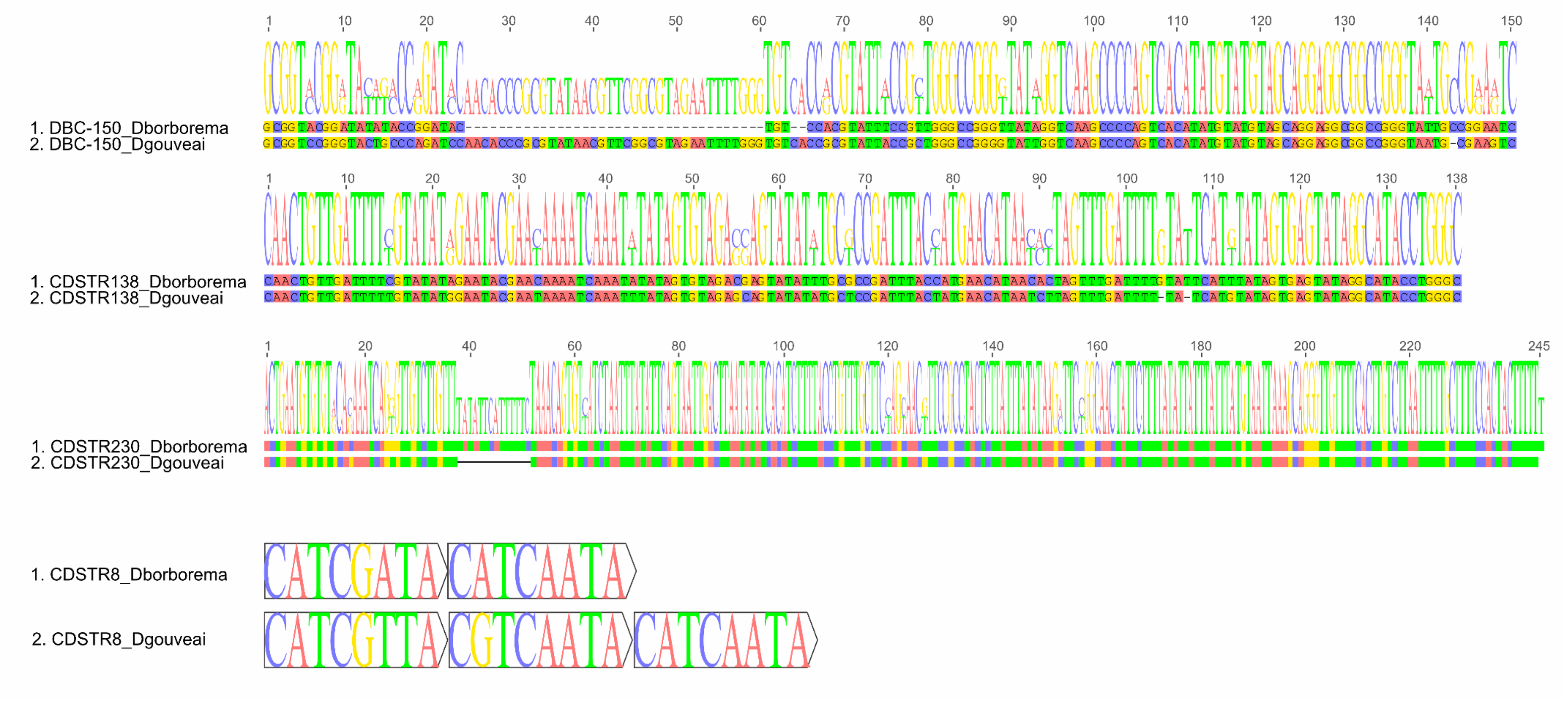


**Figure S2 -** Nucleotide alignments with satDNA consensus sequences from *D. gouveai* and *D. borborema*.
